# Familiarity with Interest Breeds Gossip: Contributions of Emotion, Expectation, and Reputation

**DOI:** 10.1371/journal.pone.0104916

**Published:** 2014-08-13

**Authors:** Bo Yao, Graham G. Scott, Phil McAleer, Patrick J. O'Donnell, Sara C. Sereno

**Affiliations:** 1 School of Psychological Sciences, University of Manchester, Manchester, United Kingdom; 2 School of Social Sciences, University of the West of Scotland, Paisley, United Kingdom; 3 Institute of Neuroscience and Psychology, University of Glasgow, Glasgow, United Kingdom; 4 School of Psychology, University of Glasgow, Glasgow, United Kingdom; University of Leicester, United Kingdom

## Abstract

Although gossip serves several important social functions, it has relatively infrequently been the topic of systematic investigation. In two experiments, we advance a cognitive-informational approach to gossip. Specifically, we sought to determine which informational components engender gossip. In Experiment 1, participants read brief passages about other people and indicated their likelihood to share this information. We manipulated target familiarity (celebrity, non-celebrity) and story interest (interesting, boring). While participants were more likely to gossip about celebrity than non-celebrity targets and interesting than boring stories, they were even more likely to gossip about celebrity targets embedded within interesting stories. In Experiment 2, we additionally probed participants' reactions to the stories concerning emotion, expectation, and reputation information conveyed. Analyses showed that while such information partially mediated target familiarity and story interest effects, only expectation and reputation accounted for the interactive pattern of gossip behavior. Our findings provide novel insights into the essential components and processing mechanisms of gossip.

## Introduction

Gossip is an exchange of evaluative information about an absent third party [1]. It is a social behavior, universal across culture, language, status, intelligence, gender, and age. Researchers have suggested that gossip plays an important role in managing our social dynamics – it can bond social groups together [2], communicate unwritten group norms [3], or forge social comparisons [4], for instance, in order to enhance one's status [5].

Although we frequently engage in gossiping in our daily life, the psychological antecedents of this social activity are still largely underspecified. By its nature, gossip is particular, heterogeneous in content, and relatively short-lived. Consequently, gossiping behavior is difficult to experimentally manipulate and measure. Research on gossip is often a trade-off between external validity and experimental control. For example, while research into workplace gossip is externally valid, it necessarily involves non-parametric, qualitative accounts of workplace interactions (e.g., [6]). On the other hand, studies exploring the role of gossip in public goods games create more artificial, experimentally-controlled environments for gossip to occur but cannot systematically manipulate gossip itself (e.g., [7]).

Understanding the nature of gossip hence can benefit from more parametric experimental research [1]. For example, what counts as a good piece of gossip? The working definition of gossip – an exchange of evaluative information about an absent third party [1] – hypothetically characterizes gossip across the dimensions of its target and its content. To our mind, a good piece of gossip should be judged as information that is worthy of being passed on (i.e., its content is interesting) to those who are well-placed to appreciate its content (i.e., the target is familiar to them). Nevertheless, designing materials which meet these criteria is difficult given the local, topical, and specific nature of gossip as communication. A properly controlled yet valid experiment requires interesting information which can be shared but that is not tied to knowledge of an individual's immediate, local networks. To our knowledge, no such study has been implemented. We carried out two experiments with the aim of systematically parameterizing gossip by defining some of the key factors that determine perceivers' responses to gossip. Specifically, we postulated that two of the basic factors that engendered gossip were perceivers' knowledge of the target (Familiarity) and whether the information presented about the target was engaging (Interest). In Experiment 1, participants read scenarios about celebrities or fictitious characters and indicated how likely it was that they would share this information with friends. Celebrities were chosen as familiar targets of potential gossip because they are both widely recognized and (vicariously) intimately known. Furthermore, there is evidence that we tend to share emotional social information with others (e.g., [8]–[10]). It has also been demonstrated that gossip plays a key role in maintaining our reputation systems [11], [12]. In Experiment 2, we additionally probed emotion, expectation, and reputation aspects of the scenarios and examined how these influenced potential gossiping behavior.

## Experiment 1

All participants in Experiment 1 and 2 gave written informed consent and the experimental procedures were approved by the College of Science and Engineering Ethics Committee at the University of Glasgow.

### Participants

Twenty native English speaking members of the University of Glasgow community (18 females; age 18–29, *M* = 22, *SD* = 2) participated in this study. For inclusion in the experiment, they completed a questionnaire measuring their familiarity with UK and US celebrities. Participants indicated their opinions toward 200 names (100 celebrities, 100 fictitious non-celebrities) using a 7-point scale (−3 = *I genuinely dislike them*; −2 = *I quite dislike them*; −1 = *I somewhat dislike them*; 0 = *I know their name, but it's just a name*; 1 = *I somewhat like them*; 2 = *I quite like them*; 3 = *I'm a real fan of them*; and an additional category, *I don't recognize their name*, which we also coded as 0). Absolute values (i.e., magnitude) of these ratings were taken, treating “likes” and “dislikes” as being equally familiar with the target. Participants were “familiar” (≥1) with 91% of the celebrities (*SD* = 7%) and were “unfamiliar” ( = 0) with 99% of the non-celebrities (*SD* = 4%). Three additional participants having low familiarity of celebrities (“familiar” proportion <70%) were excluded from further testing.

### Design and Materials

A 2 (Target Familiarity: Celebrity vs. Non-celebrity) ×2 (Story Interest: Interesting vs. Boring) within-participants design was implemented in fictitious short passages. The first sentence introduced either the Celebrity or Non-celebrity and the remaining two sentences, used with both versions, were deemed either Interesting or Boring. **Table 1** provides an example stimulus set in which each version of the first sentence can be used in combination with alternative versions of the remaining sentences, giving rise to the four experimental conditions. Each participant read only one of the four possible conditions of each story. One-hundred such quadruples of stories were generated, yielding four counterbalanced lists (25 items per condition) presented to equal numbers of participants.

**Table 1:** Example Set of Four Fictitious Stories that Vary as a Function of Target Familiarity and Story Interest.

**Sentence 1**	
**Celebrity**	*Barack and Michelle Obama visited the Bastille during a diplomatic visit to Paris.*
**Non-celebrity**	*David and Theresa O'Hara visited the Bastille during a diplomatic visit to Paris.*
**Sentences 2 and 3**	
**Interesting**	*Afterwards, they had to take their kids to McDonald's because they refused to eat French food. The other diners were very amused by their presence in the fast-food chain.*
**Boring**	*Their kids were given a personal tour and the history was explained by a local tour guide. They took a lot of photos at the Bastille and later at the Eiffel Tower.*

*Note*: Each version of Sentence 1 (Celebrity, Non-celebrity) can be combined with alternative versions of Sentences 2 and 3 (Interesting, Boring) to yield different stories in the four experimental conditions. The celebrities chosen were of a variety of backgrounds including musicians, movie stars, TV presenters, media tycoons, models, politicians, royalties, footballers, celebrity chefs, etc. Some of them appear as couples and some of them appear on their own in our stimuli.

### Apparatus and Procedure

Participants were instructed that they would be asked to appraise recent stories about people. Stories were displayed on a computer screen and ratings were obtained from a 4-key response pad. After reading each scenario, participants rated (a) how likely it was that they would tell this information to friends (1 = *very unlikely*; 2 = *somewhat unlikely*; 3 = *somewhat likely*; and 4 = *very likely*), followed by (b) how interesting they considered the information itself to be, irrespective of the characters involved (1 = *very boring*; 2 = *somewhat boring*; 3 = *somewhat interesting*; and 4 = *very interesting*). We will refer to these as the “gossip” and “appeal” ratings, respectively. Upon completion, participants were debriefed as to the fictitious nature of the stories.

### Results and Discussion

We first assessed the validity of our Familiarity and Interest variables using paired-sample *t*-tests. We then conducted Familiarity×Interest analyses of variance (ANOVAs) on the “gossip” rating data. All statistical analyses were performed both by participants (*t*_1_, *F*_1_) and by items (*t*_2_, *F*_2_). The mean “gossip” ratings across conditions are presented in **Table 2**.

**Table 2:** Mean Ratings for Likelihood to Gossip as a Function of Target Familiarity and Story Interest.

Experiment 1				
	Interesting	Boring	*t*_1_(19)	*t*_2_(99)
**Celebrity**	2.79 (1.00)	1.95 (0.98)	5.44***	12.96***
**Non-celebrity**	2.05 (1.10)	1.64 (1.00)	3.71**	4.10***
***t*** **_1_(19)**	3.56**	2.79*		
***t*** **_2_(99)**	10.71***	3.77***		

Experiment 2				
	Interesting	Boring	*t*_1_(35)	*t*_2_(99)
**Celebrity**	4.79 (1.92)	2.44 (1.68)	18.08***	17.59***
**Non-celebrity**	3.48 (2.06)	1.70 (1.11)	9.78***	16.27***
***t*** **_1_(35)**	8.79***	5.60***		
***t*** **_2_(99)**	12.18***	10.25***		

*Note*: Likelihood to gossip was measured on a scale of 1 to 4 (low to high) in Experiment 1 and on a scale of 1 to 7 (low to high) in Experiment 2. Standard deviations are in parentheses. Also shown are results of follow-up contrasts to the Familiarity×Interest interaction, including *t*-values and significance thresholds (*p*<.001 = ***, *p*<.01 = **, and *p*<.05 = *).

#### Manipulation checks

For Familiarity, paired-sample *t*-tests on the absolute value of participants' initial ratings (i.e., their opinions of the 200 names) indicated significantly higher familiarity with Celebrities (*M* = 1.56, *SD* = .91) than Non-celebrities (*M* = .03, *SD* = .23) [*t*_1_(19) = 24.90, *p*<.001, Cohen's *d* = 7.62; *t*_2_(99) = 32.41, *p*<.001, Cohen's *d* = 4.55]. For Interest, paired-sample *t*-tests performed on “appeal” ratings revealed significantly higher ratings for stories deemed Interesting (*M* = 2.63, *SD* = .92) than Boring (*M* = 1.89, *SD* = .93) [*t*_1_(19) = 4.98, *p*<.001, Cohen's *d* = 1.32; *t*_2_(99) = 16.86, *p*<.001, Cohen's *d* = 2.08]. These results indicated that our manipulations were effective.

#### Likelihood to gossip

ANOVAs on “gossip” ratings revealed significant main effects of Familiarity [*F*_1_(1,19) = 12.11, *p*<.01, Cohen's *f* = .80; *F*_2_(1,99) = 74.42, *p*<.001, Cohen's *f* = .87; min*F′*(1,26) = 10.41, *p*<.01], Interest [*F*_1_(1,19) = 30.32, *p*<.001, Cohen's *f* = 1.26; *F*_2_(1,99) = 78.07, *p*<.001, Cohen's *f* = .89; min*F′*(1,36) = 21.84, *p*<.001], as well as a significant Familiarity×Interest interaction [*F*_1_(1,19) = 9.27, *p*<.01, Cohen's *f* = .70; *F*_2_(1,99) = 23.17, *p*<.001, Cohen's *f* = .48; min*F′*(1,36) = 6.62, *p*<.05]. The simple effects are reported in **Table 2**. While both Familiarity and Interest each accounted for an increased tendency to gossip, participants were significantly more likely to gossip when information was both interesting *and* involved familiar celebrities.

## Experiment 2

Experiment 1 established Familiarity and Interest as two fundamental factors that give rise to gossiping behavior. Experiment 2 further investigated the interaction between these factors. We sought, first, to replicate the results in Experiment 1 with a larger, less selective sample and, second, to assess some of the key semantic features of the stories that may govern the effects of familiarity and interest and their interaction.

### Participants

Thirty-six native English speaking members (34 females; age 19–31, *M* = 22, *SD* = 3) of the University of Glasgow Subject Pool (intranet.psy.gla.ac.uk/subject-pool/web/) participated in this study. Although we did not pre-screen participants for their familiarity with celebrities in this experiment, a degree of self-selection occurred as the study was advertised as “Celebrity Gossip”.

### Design and Materials

The design and materials were the same as in Experiment 1.

### Apparatus and Procedure

The experiment was implemented via our online testing platform at Glasgow (experiments.psy.gla.ac.uk). Each story was displayed centrally on a computer screen, followed by 6 sets of ratings each using 7-point Likert scales (see **Table S1** for the rating questions and scale labels). In addition to obtaining “gossip” ratings, the 5 remaining measures were developed to capture the extent to which readers were affected by emotion, expectation, and reputation aspects of the stories. Emotional information is typically defined as that which is high in arousal and positively- or negatively-valenced (e.g., [13], [14]). Accordingly, we measured the *arousal* and *valence* levels elicited by the stories. Expectation was assessed by story *plausibility* and *surprise* ratings. Reputational shifts of the gossip target(s) were assessed by the *change in opinion* toward the story protagonist(s). We will refer to these 5 measures as “arousal”, “valence”, “plausibility”, “surprise”, and “Δopinion”, respectively. Upon completion of the experiment, participants were debriefed as to the fictitious nature of the stories.

### Results and Discussion

#### Replicating the Familiarity and Interest effects

ANOVAs on “gossip” ratings revealed significant main effects of Familiarity [*F*_1_(1,35) = 74.10, *p*<.001, Cohen's *f* = 1.45; *F*_2_(1,99) = 197.06, *p*<.001, Cohen's *f* = 1.41; min*F′*(1,63) = 53.85, *p*<.001], Interest [*F*_1_(1,35) = 221.43, *p*<.001, Cohen's *f* = 2.52; *F*_2_(1,99) = 363.32, *p*<.001, Cohen's *f* = 1.91; min*F′*(1,80) = 137.58, *p*<.001], as well as a significant Familiarity×Interest interaction [*F*_1_(1,35) = 14.07, *p*<.01, Cohen's *f* = .63; *F*_2_(1,99) = 26.35, *p*<.001, Cohen's *f* = .52; min*F′*(1,75) = 9.17, *p*<.01]. The results replicated those of Experiment 1, showing that, while Familiarity and Interest each individually increased the likelihood to gossip, participants were significantly more likely to gossip when familiar targets were linked to interesting stories (**Table 2**, lower panel).

#### Assessing the contributions of story emotionality, expectation, and character reputation

**Figure 1:**
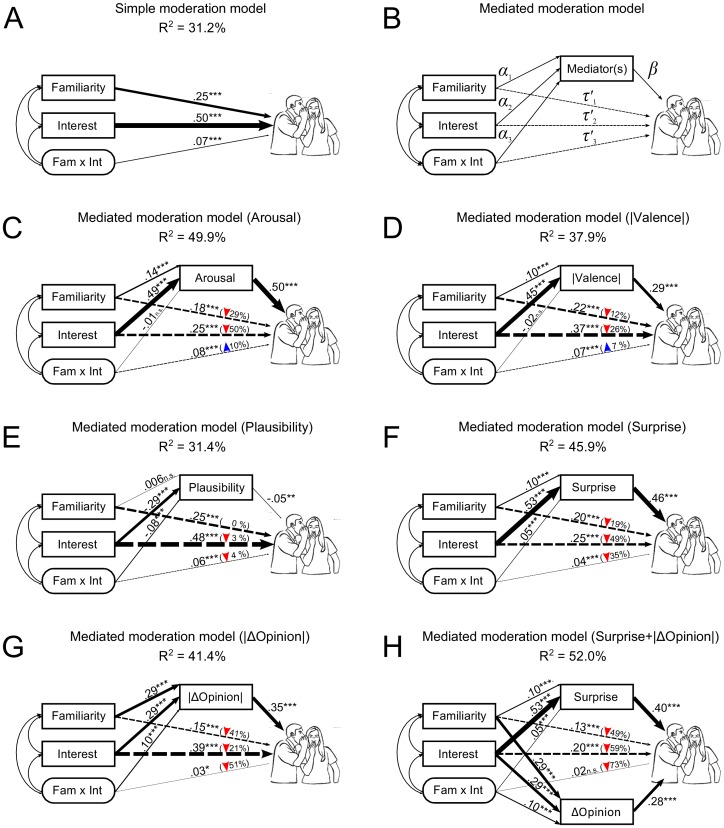
Illustrations of the Simple Moderation and Mediated Moderation Models Tested. **A**. Simple moderation model (Model 1 of Hayes, 2012). **B**. Basic mediated moderation model (Model 8 of Hayes, 2012). *α*s refer to the slope coefficients of the mediator(s) regressed on Familiarity, Interest, and their interaction. *β*(s) and *τ*'s denote the coefficients of Gossip regressed on the mediator(s) and the predictors, respectively, when both are included as simultaneous predictors of Gossip. **C**, **D**, **E**, **F**, and **G**. The mediated moderation models with Arousal, |Valence|, Plausibility, Surprise, and |ΔOpinion| as mediators, respectively. **H**. The mediated moderation model with Surprise and |ΔOpinion| as simultaneous mediators. *Note:* Coefficients are reported on their corresponding regression paths. Significance thresholds are *p*<.001 = ***, *p*<.01 = **, *p*<.05 = *, and not significant = *ns*. The percentages of direct effects explained by indirect effects are reported in parentheses on the corresponding direct paths. Downward and upward arrows indicate decreases and increases, respectively, of direct effects in comparison to the simple moderation model.

In order to better determine what accounted for the Familiarity and Interest effects and, in particular, their interaction, we tested a simple moderation model (**Figure 1A**; Model 1 of Hayes, 2012) versus a mediated moderation model (**Figure 1B**; Model 8 of Hayes, 2012). We considered 5 mediated moderation models with Arousal, Valence magnitude (|Valence|), Plausibility, Surprise, and ΔOpinion magnitude (|ΔOpinion|) as the respective mediators (**Figure 1C–1G**). We employed the bootstrapping techniques of Hayes (2012; PROCESS macro version 2.04) using 10,000 bootstrap re-samples with bias corrected and accelerated confidence intervals (at 95%, 99% and 99.9% levels) as recommended. All variables were standardized before the analyses.

The simple moderation analysis confirmed the pattern of effects from the ANOVA, with significant main effects of Familiarity and Interest as well as the two-way interaction (**Figure 1A**). The mediated moderation analyses revealed that the main effects of Familiarity and Interest were both *partially* mediated by all five mediators (see “conditional indirect effects” in **Table 3**. The effects associated with the mediator Plausibility, however, were relatively weak (for main effects, the interaction, and the zero-order regression; **Figure 1E**) as compared to those of the other mediators (**Figure 1C, 1D, 1F, and 1G**). Moreover, including Plausibility in the model resulted in only a 0.2% increase in the variance explained (R^2^) as compared with the original simple moderation model (**Figure 1E** vs. **1A**). This may be due in part to the fact that Plausibility judgments could be based on the story plots, themselves, without considering specific characters and their behaviors. It suggests that this variable may not be as “good” a predictor of Gossip and should be modified or omitted in future investigations. Nevertheless, in terms of the main effects of Familiarity and Interest, moderated mediation analyses revealed that the likelihood to gossip was significantly accounted for by partial transmission via the separate mediators of Arousal, |Valence|, Plausibility, Surprise, and |ΔOpinion|.

**Table 3:** Beta values for the Indirect (Mediation) Effects on the Likelihood to Gossip of Familiarity, Interest, and their Interaction.

	Conditional indirect effects of Fam on Gossip at values of Int		Conditional indirect effects of Int on Gossip at values of Fam		
Mediator	Low Int (−SD)	High Int (+SD)	Low Fam (−SD)	High Fam (+SD)	Indirect effect of Fam×Int
**Arousal**	0.078***	0.065***	0.256***	0.242***	−.007 *ns*
**|Valence|**	0.035***	0.026***	0.134***	0.125***	.004 *ns*
**Plausibility**	−0.004**	0.003**	0.010**	0.017**	.004**
**Surprise**	0.023**	0.072***	0.217***	0.267***	.025***
**|ΔOpinion|**	0.065***	0.137***	0.068***	0.139***	.036***

*Note:* Fam = Familiarity, Int = Interest. Significance thresholds: *p*<.001 = ***, *p*<.01 = **, and not significant = *ns*.

With respect to the Familiarity×Interest interaction, our analyses indicated that only Plausibility, Surprise and |ΔOpinion| reliably mediated the effect (see final column of **Table 3**). We then examined if the interaction effect could be fully accounted for in a multiple mediated moderation model. Given the weak effect size of Plausibility (see discussion above), we first included Surprise and |ΔOpinion| as parallel mediators in the model for a simpler interpretation of the results (**Figure 1H**). The analysis showed that the direct effect of the interaction (*τ_3_* = .07; **Figure 1A**) became non-significant (*τ′_3_* = .02; **Figure 1H**). This indicates that the Familiarity×Interest interaction is already *fully* mediated jointly by Surprise and |ΔOpinion| (the pattern remained almost identical when the third mediator Plausibility was additionally included).

Overall, while Familiarity and Interest each accounted for an increased likelihood to gossip, these effects were also partially explained by the mediators of Arousal, |Valence|, Plausibility, Surprise, and |ΔOpinion|. In contrast, the Familiarity×Interest interaction could be fully mediated by Surprise and |ΔOpinion|.

## Conclusions

We investigated Familiarity and Interest as two key factors that robustly predicted gossip behavior. Our data showed that individuals were more likely to gossip about familiar versus unfamiliar targets and interesting versus boring information. In addition, these effects could be accounted for in part by differences in the magnitude of emotions, expectations, and reputational shifts elicited by the stories and their characters. Critically, however, we found that individuals were even more likely to gossip when a story they read united a familiar person with an interesting scenario. Further analyses revealed that these elevated levels of gossip were selectively linked to expectational and reputational aspects of the stories. One interpretation is that the reader's surprise reflects an initial reaction to a specific character within an unpredictable story, in particular, in the wider context of how that target's behavior relates to social norms. This response serves to trigger subsequent evaluation and reappraisal of the target. The optimal conditions for gossip to occur, then, will be when individuals encounter stories about familiar targets involved in interesting, socially-relevant situations.

The central feature of our approach is that experimental control is achieved by directly manipulating and measuring key variables affecting the impact of gossip on the individual. Given the ascendancy of celebrities in our society [16], [17], the use of celebrities as proxy third parties for potential gossip is procedurally valid. Moreover, it can facilitate the exploration of the role of gossip in social reputation systems [5], [11]. It is worth noting that we tend to gossip chiefly about our day-to-day contacts (e.g., friends, family, colleagues, etc.). Because we have non-overlapping sets of acquaintances with distinctive habits, however, implementing appropriate experimental control over such stimuli across participants would be challenging. Although we believe that gossip about such people would operate similarly to that with celebrity targets, future research should nonetheless begin to investigate the relationship between familiarity and interest in interpreting stories about our non-celebrity, everyday acquaintances.

The recent past has witnessed not only the increased accessibility to the established media, but the emergence and infectious spread of social media, as well as an ever more present intelligent monitoring of personal communications. In this context, it is thus pertinent to establish the factors influencing gossip processing and to recognize the role of gossip in reputation management (e.g., [18]). In these ways, our study provides a behavioral foundation for more sophisticated research into the cognitive processes and neural correlates underlying this pervasive socio-linguistic phenomenon.

## Supporting Information

Table S1 **Story Ratings.**
(DOCX)
