# Supplementary material for: Familiarity with Interest Breeds Gossip: Contributions of Emotion, Expectation, and Reputation
Source: PLoS One. 2014 Aug 13;9(8):e104916. doi: 10.1371/journal.pone.0104916 (PMC4132070; doi:10.1371/journal.pone.0104916)
Supplement: Table S1 — Story Ratings. (DOCX) [file pone.0104916.s001.docx]

**Supplemental Online Materials**

**Table S1**

**Story Ratings**

| **Measure** | **Question** | **Labels** |
| --- | --- | --- |
| **Arousal** | How EMOTIONALLY AROUSING do you find the story? | 1=Not at all arousing; 7=Extremely arousing |
| **Valence** | How POSITIVE or NEGATIVE do you find the story? | -3=Very negative; 0=Neutral; 3=Very positive |
| **Plausibility** | How PLAUSIBLE do you find the story? | 1=Not at all plausible; 7=Extremely plausible |
| **Surprise** | How SURPRISING do you find the story? | 1=Not at all surprising; 7=Extremely surprising |
| **ΔOpinion** | If you know the person(s) mentioned in the story, how does this information CHANGE YOUR OPINION about him/her/them? | -3=Much worse; 0=No change; 3=Much better; 0=I don’t know them |
| **Gossip (DV)** | How LIKELY is it that you would TELL SOMEONE this story? | 1=Would never; 7=Would definitely |

*Note*: Each story was rated on the 6 measures in the order listed. The associated questions and labels for each rating scale are also indicated.
